# Subconjunctival and Orbital Silicone Oil Granuloma (Siliconoma) Complicating Intravitreal Silicone Oil Tamponade

**DOI:** 10.1155/2014/686973

**Published:** 2014-04-24

**Authors:** Jung Hye Lee, Yoon-Duck Kim, Kyung In Woo, Mingui Kong

**Affiliations:** ^1^Department of Ophthalmology, Kim's Eye Hospital, Myung-Gok Eye Research Institute, College of Medicine, Konyang University, Seoul 150-034, Republic of Korea; ^2^Department of Ophthalmology, Samsung Medical Center, Sungkyunkwan University School of Medicine, 50 Irwon-dong, Gangnam-gu, Seoul 135-710, Republic of Korea

## Abstract

A 30-year-old male, who underwent previous pars plana vitrectomy and silicone oil tamponade due to endogenous endophthalmitis originated from Klebsiella liver abscess, was referred for evisceration. At 2 months after vitrectomy with silicon oil tamponade, conjunctival chemosis and ocular pain were aggravated. Diffuse eyelid swelling and large subconjunctival mass with lipid droplets were noted. On MRI examination, subconjunctival mass and intra- and extraconal orbital mass around superior rectus muscle were observed. Excision of subconjunctival and orbital mass was performed. Histopathologic examination showed multiple silicone oil vacuoles surrounded by foreign body giant cells and fibrosis, which confirmed silicone oil granuloma. In a patient with suspicious melting sclera in diseases such as endophthalmitis, large silicone oil granuloma may be complicated in a rapid fashion after intravitreal silicone oil tamponade due to silicone oil leakage.

## 1. Introduction

Silicone oil has been widely used for decades in complex vitreoretinal surgeries. Although it has been known to be inert material, a number of complications such as cataract, glaucoma, and retinal toxicity have been reported [1]. However, there are few extraocular complications associated with silicone oil leakage following retinal surgery with silicone oil tamponade [2–4].

To our knowledge, there have been no previous reports of early onset of large granuloma caused by extraocular migration of intravitreal silicone oil. We report subconjunctival and orbital silicone oil granuloma that resulted from the leakage of intravitreal silicone oil in a patient with endogenous endophthalmitis.

## 2. Case Report

A 30-year-old male suddenly developed left eyeball pain and visual loss with fever and chilling sensation. He was diagnosed as endogenous Klebsiella endophthalmitis that originated from liver abscess. He underwent pars plana vitrectomy and silicone oil tamponade (Oxane 5700, Bausch & Lomb, Waterford, Ireland, UK) in the left eye for severe endogenous endophthalmitis. The infection had been calmed down after the surgery until the eyelid swelling and chemosis were aggravated at 2 months postoperatively. He was referred to oculoplastic clinic for evisceration under the impression of uncontrolled endophthalmitis.

**Figure 1:**
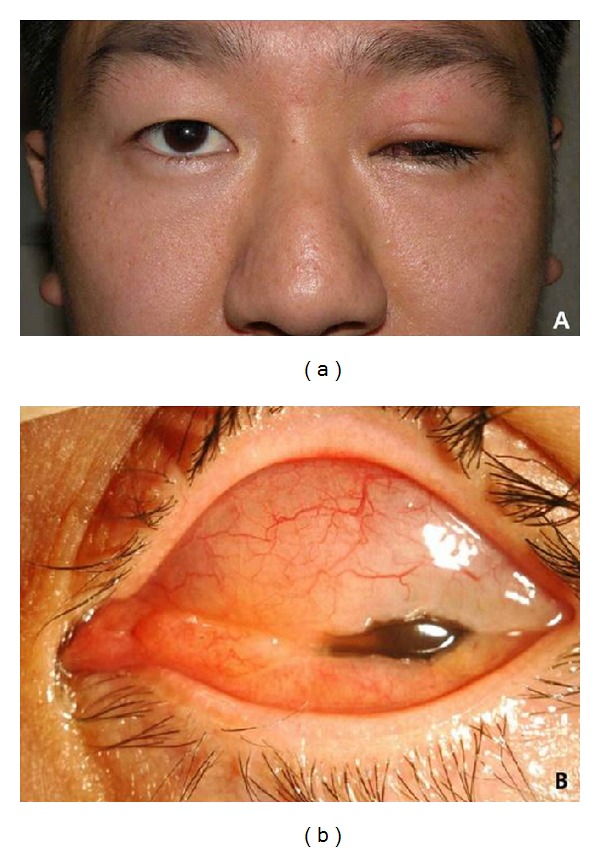
Clinical photographs at presentation. (a) Left upper eyelid shows diffuse erythematous swelling with complete ptosis. (b) Upper bulbar subconjunctival mass with underlying multiple small transparent lipid droplets of silicone oil.

On ophthalmologic examination, he had no light perception in the left eye. The intraocular pressure was 14 mmHg in the right eye and 9 mmHg in the left eye. And left upper eyelid showed diffuse swelling and redness with complete ptosis (Figure 1(a)). Large upper subconjunctival mass was covering the cornea, in which underlying multiple small transparent lipid droplets were observed under slit lamp examination (Figure 1(b)). Cornea was clear and dense fibrotic membranous tissues were noted in anterior chamber.

**Figure 2:**
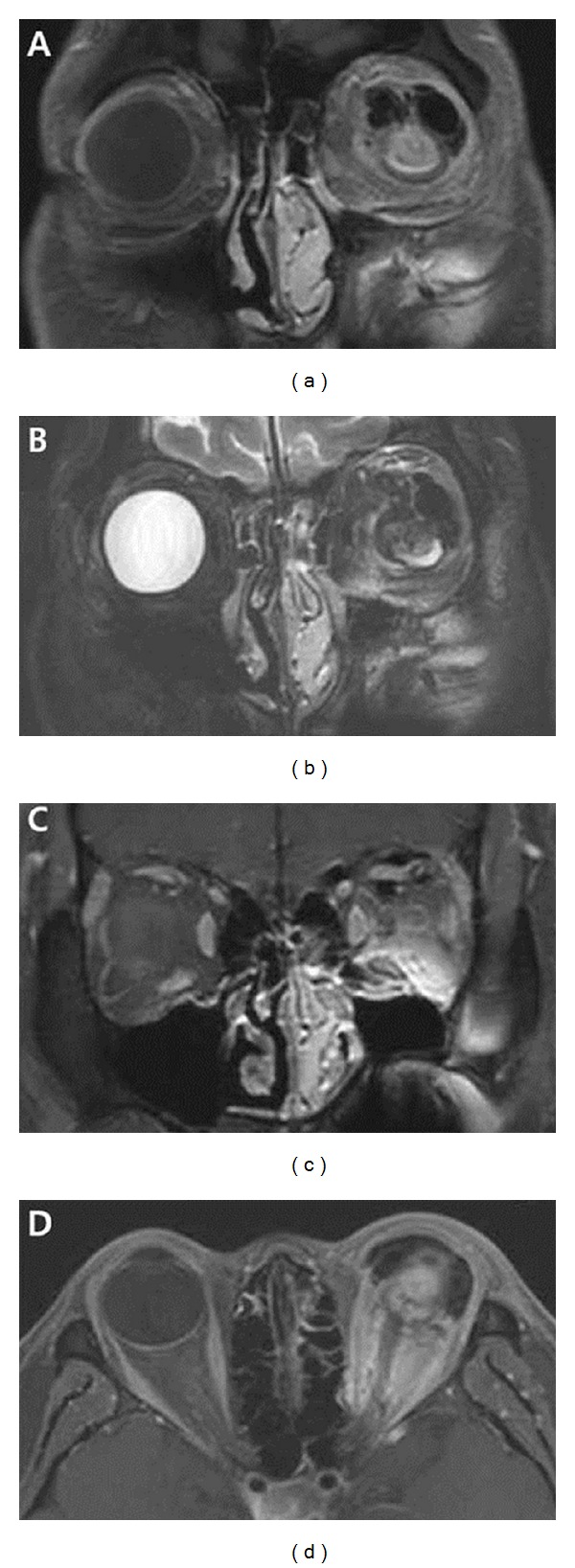
Magnetic resonance image (MRI). (a), (b) T1- and T2-weighted images reveal a large septate cystic mass in subconjunctival and orbital space with low signal intensity. (c), (d) The mass is located in the superior orbit accompanied by inflammatory reaction showing heterogenous enhancement in Gd-enhanced T1-weighted images.

Orbital magnetic resonance image (MRI) examination showed shrinkage of the eyeball and large subconjunctival and orbital mass. The mass was located in superior part of the orbit between levator muscle and eyeball, surrounding superior rectus muscle. It demonstrated septate cystic form with low signal intensity in T1- and T2-weighted image and heterogenous enhancement in Gd-enhanced image (Figure 2).

**Figure 3:**
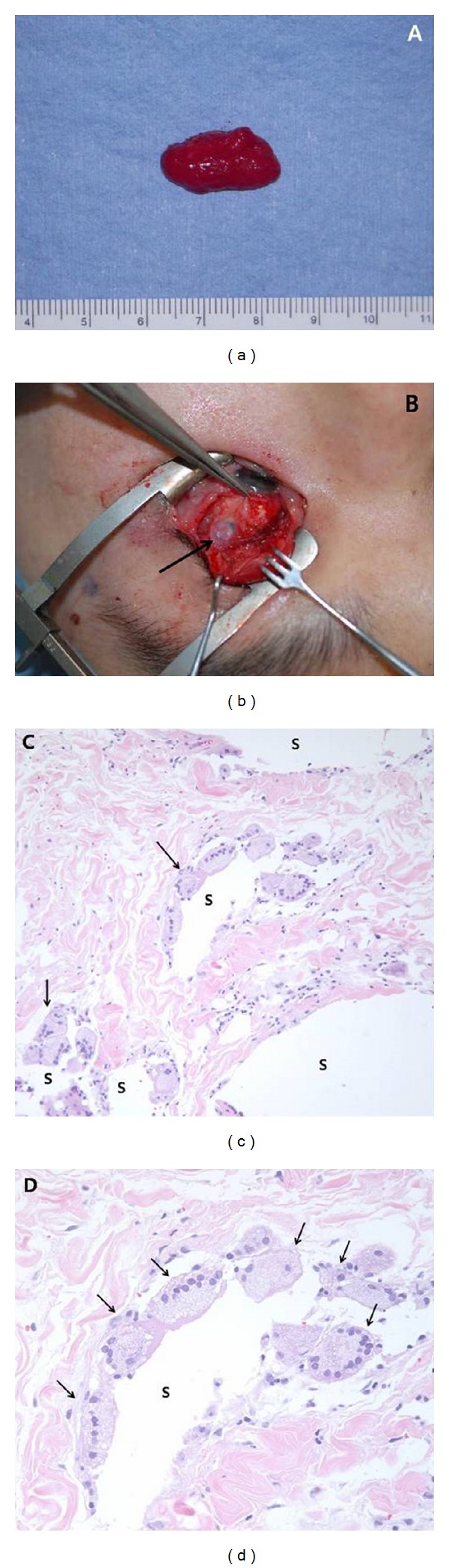
Excision of silicone oil granuloma and histopathology of excised mass. (a) Removed mass which was caused by silicone oil leakage. (b) Melting focus located at 1 o'clock around equator of eyeball which was already healed. Brownish uveal tissue (arrow). (c), (d) Multiple small and large lipid droplets which are silicone oil material (s) surrounded by foreign body giant cells (arrows) with less prominent fibrosis (Hematoxylin and Eosin stain, ×200 and ×400, resp.).

The authors performed excision of subconjunctival and orbital mass. Large subconjunctival mass was observed under the entire upper bulbar conjunctiva. The conjunctiva was separated from the underlying mass by blunt dissection. Granulomatous mass was removed (Figure 3(a)) and the remaining silicone oil surrounding superior rectus muscle was squeezed with cotton tips. Silicone oil itself was visible around the eyeball. The melted sclera was noted at 1 o'clock around the equator of eyeball which was already healed and underlying brownish uveal tissue was visible (Figure 3(b)).

Histopathology revealed silicone oil globules with inflammatory cellular infiltration. Multiple small and large lipid droplets (silicone oil) surrounded by foreign body giant cells were noted (Figures 3(c) and 3(d)). The fibrosis was less prominent compared with previous siliconomas formed in breast or other parts of body.

At 6 months after the surgery, the patient showed no evidence of recurrence. The patient was comfortable with no pain and showed good cosmesis wearing the prosthesis.

## 3. Discussion

Silicone oil is an established tamponade in treating complex vitreoretinal diseases such as retinal detachments, proliferative diabetic retinopathy associated with tractional retinal detachment [5]. It is also primarily used in eyes with severe trauma or acute endophthalmitis to stabilize the retina and inhibit the proliferative activity [6]. Intravitreal silicone oil is intended to be removed after several weeks to months due to its anterior or posterior segment complications and possible extraocular problems such as cataract, glaucoma, corneal decompensation, optic neuropathy, retinal toxicity, or extraocular migration. The presence of extraocular silicone oil is a rare complication of intravitreal silicone oil tamponade. Few reports demonstrated silicone oil migration into the subconjunctival space, eyelid, optic nerve, or brain [2–4, 7, 8]. There have been 3 cases of silicone oil intrusion in the upper eyelid 1, 8, and 19 years after vitreoretinal surgery [2, 8], and Nazemi et al. [4] reported 1 case of subconjunctival silicone oil granuloma leaked through Ahmed glaucoma valve. Srinivasan et al. [9] described 2 cases of episcleral granulomas adjacent to vitrectomy entry sites after silicone oil tamponade. These previous studies usually showed delayed manifestation of extraocular silicone oil from 1 to 19 years. And silicone oil leakage occurred through the sclerotomy sites or transscleral implant.

In the present study, silicone oil granuloma developed rapidly within 2 months through the melted sclera in a patient who underwent silicone oil tamponade for endogenous Klebsiella endophthalmitis originated from liver abscess. Endogenous Klebsiella endophthalmitis has generally poor prognosis in which most patients result in either no light perception or evisceration or enucleation [10]. Although it is known that early intervention of vitrectomy with silicone oil tamponade is of much benefit to the vision and the stabilization of retina, sclera could be weakened by rapidly progressing intraocular infection and silicone oil may be leaked to extraocular space. In this patient, the fibrotic response was not severe which implies that the lesion progressed rapidly with the acute inflammatory reaction according to the histopathologic results.

To our knowledge, there have been no previous reports of early onset of silicone oil granuloma which was caused by large amount of silicone oil leakage through the melted sclera. Granulomatous inflammation associated with silicone oil leakage can develop rapidly progressing subconjunctival and orbital mass in patients with weakened sclera. In view of this case, surgeon should be prudent to perform silicone oil tamponade in a patient with suspicious melting sclera such as severe or uncontrolled endophthalmitis.
